# The red flour beetle *T. castaneum*: elaborate genetic toolkit and unbiased large scale RNAi screening to study insect biology and evolution

**DOI:** 10.1186/s13227-022-00201-9

**Published:** 2022-07-19

**Authors:** Martin Klingler, Gregor Bucher

**Affiliations:** 1 Department of Biology, Friedrich-Alexander-Universität Erlangen-Nürnberg (FAU), Staudtstr. 5, 91058 Erlangen, Germany; 2 Johann-Friedrich-Blumenbach-Institut, GZMB, University of Göttingen, Justus-von-Liebig-Weg 11, 37077 Göttingen, Germany

**Keywords:** Insect model system, Arthropod model system, Coleoptera, Evolution of development, Physiology, Behavior, Pest control, Systemic RNAi, Genome-wide screen, CRISPR genome editing

## Abstract

The red flour beetle *Tribolium castaneum* has emerged as an important insect model system for a variety of topics. With respect to studying gene function, it is second only to the vinegar fly *D. melanogaster*. The RNAi response in *T. castaneum* is exceptionally strong and systemic, and it appears to target all cell types and processes. Uniquely for emerging model organisms, *T. castaneum* offers the opportunity of performing time- and cost-efficient large-scale RNAi screening, based on commercially available dsRNAs targeting all genes, which are simply injected into the body cavity. Well established transgenic and genome editing approaches are met by ease of husbandry and a relatively short generation time. Consequently, a number of transgenic tools like *UAS/Gal4, Cre/Lox*, imaging lines and enhancer trap lines are already available. *T. castaneum* has been a genetic experimental system for decades and now has become a workhorse for molecular and reverse genetics as well as in vivo imaging. Many aspects of development and general biology are more insect-typical in this beetle compared to *D. melanogaster*. Thus, studying beetle orthologs of well-described fly genes has allowed macro-evolutionary comparisons in developmental processes such as axis formation, body segmentation, and appendage, head and brain development. Transgenic approaches have opened new ways for in vivo imaging. Moreover, this emerging model system is the first choice for research on processes that are not represented in the fly, or are difficult to study there, e.g. extraembryonic tissues, cryptonephridial organs, stink gland function, or dsRNA-based pesticides.

## Natural habitat and life cycle

The kitchen is a great place to start for collecting *Tribolium castaneum*. As a stored product pest, sampling "wild" populations of *Tribolium* means collecting in the house, or in flour processing or storage industries. What was the natural habitat of these synanthropic beetles before the emergence of human agriculture and grain processing? We don't know! Some speculations about the origin of *T. castaneum* (including ancient Egyptian tales) can be found in a recent review that covers current and classical *T. castaneum* work in population genetics, ecology and evolution [1]. Here, we focus on *T. castaneum* as an experimental system for molecular and functional analysis of insect genes.

**Fig. 1:**
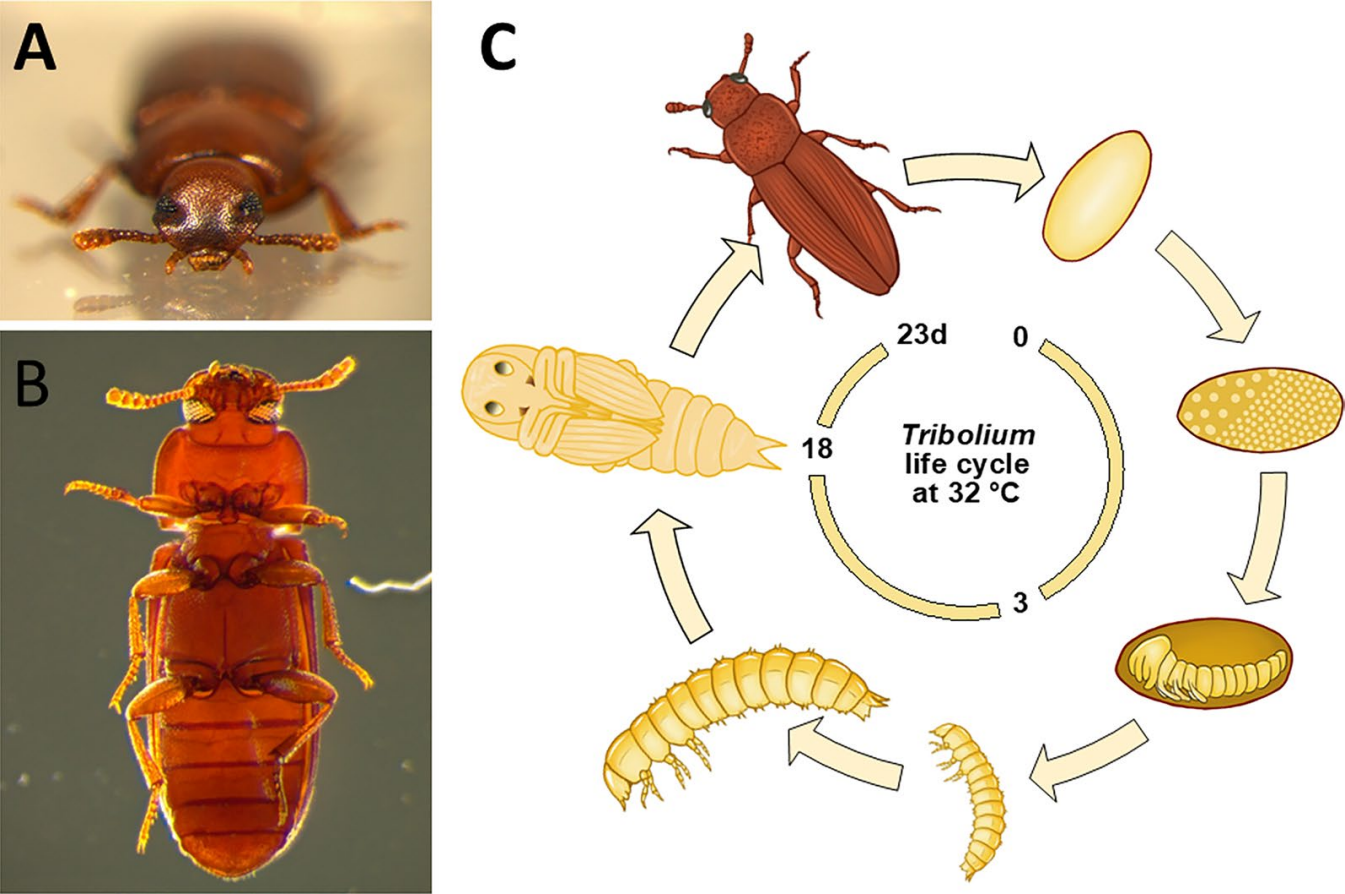
The life cycle of *T. castaneum*. **A** Face-to-face with a wild type *Tribolium castaneum*. **B** Ventral view of a male under dark field illumination. White eyes (*vermilion* mutant) allow for sensitive detection of transgene eye markers. The size of an adult beetle is 3.5 mm (length). **C** Life cycle of *Tribolium:* eggs are laid into the substrate (flour) and embryonic development takes 3 days at 32 °C. Only two out of a variable number of larval stages (ca. 7) are depicted. The *pupa libera* allows visual inspection of external structures facilitating phenotypic studies of metamorphosis. Female beetles need a few days after hatching until they start laying eggs, which they continue for 3–4 months (drawings not to scale). Life cycle sketch by Benjamin Schwarz

As a typical holometabolous insect, *T. castaneum* develops through several larval stages (usually 7, but 5 or 6 when starved [2, 3], and up to 11 instars based on some anecdotal accounts) followed by metamorphosis (Fig. 1). Its embryonic developmental comfort zone is between 22 and 32 °C, lasting 7 days at 25 °C and 3 days at 32 °C [3]. Likewise, development from egg to adult speeds up with temperature from 74 days at 22.5 °C to about 23 days at 32 °C, which is short enough for large scale genetic experiments.

## Lab culture

*T. castaneum* is easy to culture: it only requires organic (insecticide free) flour enriched with yeast powder (5%) for protein and vitamins. For stock keeping, whole wheat flour is recommended as it contains additional nutrients. Only for egg collection, the beetles are kept on white flour (of which "instant flour" or "doppelgriffiges Mehl" are easier to sieve). No bad odors need to be endured by *T. castaneum* workers, and unlike *Tenebrio*, the familiar "meal worm" sold in pet shops, no fresh vegetables need to be added. Flour beetles don't need to drink as they derive all their body fluids from dry matter: burning starch produces water. Beetles are best kept in incubators at a humidity between 40 and 60% and with an opening for supply of fresh air. Humidity should not drop below 30% while the upper limit is set by the emergence of mold in the flour. Beetles live up to a year but egg-laying rate is high only in the first 3–4 months.

Cultures can be protected from mites using dense foam stoppers or by covering ventilation openings with a fine gauze (100 µm mesh size). Cultures infected with microorganisms transmitted via flour can be "sterilized" by collecting eggs from the flour (using 300 µm sieves), suspending them in 3% formaldehyde for 15 min, collecting them on gauze and rinsing with water, and then placing the gauze on top of fresh flour (eggs facing down). Small stocks (50–200 animals) are kept in vials (e.g. typical fly vials with 5 cm diameter) with foam stoppers. Larger populations, e.g. for collection of eggs or animals to be injected, are kept in boxes of about 25 cm edge length with an opening for air exchange of around 5 cm diameter.

During experiments, beetles are usually kept at 30–32 °C to speed up development and for obtaining strongest RNAi phenotypes. Higher temperatures reduce embryonic viability. At 25 °C the developmental time roughly doubles compared to 32 °C. For stock maintenance the temperature can be reduced to 22–23 °C, where development from egg to adult lasts ca. 74 days. To ensure that synchronized animals of all stages are available at any time, a working stock can be kept in the following way: adult beetles are kept at 25 °C in large boxes on white flour supplemented with yeast. Eggs are collected twice a week from this culture and transferred to another box with fresh whole wheat flour. Transfer of eggs to new flour (instead of moving the adults to a new box) minimizes the transmission of parasites via feces. To allow for synchronized development within one batch, the culture should not be too dense. Every 3–4 months the original stock is replaced by young beetles.

Stock keeping of transgenic strains requires little work. For mutants or transgenic strains kept in heterozygous condition, we select individuals carrying a visible marker once or twice a year; 20–50 beetles are transferred to new flour at least every 6 months and fresh flour is added to those cultures periodically. This way, one person with 50% working time can keep several hundreds of transgenic stocks. For stable homozygous lines, checking the eye marker may be done even less frequently.

Developmental stages can be separated using commercially available metal wire sieves of different mesh sizes: 800 µm mesh separates adults, pupae and late larvae from whole wheat flour (pre-sieved with 700 µm mesh) and from younger stages; 500 µm retains 5th instar larvae and older stages; 300 µm separates eggs from white flour (pre-sieved with 250 µm mesh). A vacuum cleaner can be used to clean the sieves and other tools, which are then dry sterilized at 70 °C to prevent contamination of stocks with extraneous eggs or larvae.

Frequent work with flour and beetles can lead to allergies. Therefore, wearing FFP1 masks, or using a chemistry hood, is highly recommended for any work involving sieving of flour.

For dsRNA injection into females or for single pair matings, sexing is required. Animals are most easily sexed at the pupal stage by external genital anlagen (larger in females). Adult males can be identified by the presence of a gland on the femur of the first pair of legs. Animals can be sorted using sprung steel tweezers. Movement of adults and larvae can be reduced by placing them in a glass petri dish on ice, or through anesthesia with carbon dioxide using standard fly equipment.

## Major interests and research questions

*T. castaneum* has traditionally been used for developmental, ecological, behavioral and physiological studies [1, 3, 4]. In the 1960s and 70 s, a large number of mutants were recovered and studied [5]. This included for instance mutations of Hox genes, including a deficiency in the Hox cluster transforming all segments to antennal fate [6–10]. Much of the early work was summarized by Alexander Sokoloff in his three volume book [3–5]. Molecular genetics of *T. castaneum* took off in the 1990s e.g. with work in the Denell lab on the Hox complex [11, 12]. Pioneering comparisons of expression and function of orthologs of fly segmentation genes in Germany and the US [13–15] started off a major effort on macroevolutionary comparisons of patterning gene functions between fly and beetle [16–25].

In many respects the development and the biology of *T. castaneum* is more typical of insects than that of *D. melanogaster* [26, 27]. Recently, the gene sets identified to be required in the same developmental processes in *D. melanogaster* versus *T. castaneum* were systematically compared based on data from FlyBase and from the large scale RNAi screen *iBeetle* [28]. Only half of the genes involved in a given biological process were detected in both fly and beetle, while the other half were detected in only one species. This divergence probably reflects both biological divergence and technical differences in the screening approaches. These data underline the necessity of additional model systems even for well-studied processes [29]. Increasingly, *T. castaneum* is also used to study basic biological questions for which other model systems are less well suited.

### Embryo polarity and patterning

The genetic underpinnings of early development are best understood in *D. melanogaster*. However, this species differs from insect-typical embryogenesis in several aspects. For instance, most cells of the blastoderm contribute to the embryo, and all segments emerge almost at the same time (long germ development) while in most other insects, a large part of the blastoderm is extraembryonic and posterior body segments are added sequentially from a posterior segment addition zone (short germ development) [26, 30]. The homologues of most developmental genes originally identified in *D. melanogaster* turned out to play similar roles in *T. castaneum*. However, quite a few of them serve different functions, demonstrating that evolution of early developmental processes is surprisingly unconstrained. For example, pair-rule genes are expressed in double-segmental stripes as in *D. melanogaster* but appear one after the other in the *T. castaneum* segment addition zone, driven by a segmentation clock [18, 31, 110] regulated by a frequency or speed gradient [32, 33]. Since primary pair rule genes are part of the segmentation clock, their knock-down leads to complete segmentation breakdown, instead of the pair rule phenotypes known from *D. melanogaster*. While later-active segmentation genes such as segment polarity and Hox genes seem to be functionally more conserved [34, 35] the functions of gap gene orthologs have diverged [17, 20, 36, 37]. Interestingly, some genes have important functions in patterning the beetle body axis, which were not know from flies [38–40]. The early gradients initiating embryonic axis formation are unexpectedly different from *D. melanogaster*, both along the dorso-ventral [23, 24, 41], and the anterior-posterior axes. For instance, Wnt signaling is involved in early axis formation in most animals including vertebrates but not in *D. melanogaster*. In *T. castaneum*, it was found that anterior localization of the mRNA of a Wnt inhibitor led to early asymmetry of Wnt signaling involved in axis formation and proper anterior and posterior development [19, 21, 22].

Analyses of genes with conserved functions have also revealed new insights. For instance, Hox gene mutations in *T. castaneum* demonstrated a largely conserved function [8, 10] but they are located on an undivided Hox complex (similar to most animals but not *D. melanogaster*) [6, 42]. Analyses of embryonic phenotypes of HOX gene mutations and the HOX cluster deletion unequivocally demonstrated that the "ground state" of insect segment identity is antennal. This hypothesis had previously been formulated in flies based on phenotypes observed after clonal analysis in adults. However, the respective transformations had not been found in fly embryos [43]. Studying the regulation and function of the Hox cluster in *T. castaneum* may shed light on the reason for the conserved linkage of animal Hox clusters, contrasting the split observed in flies.

### Morphogenetic movements and extraembryonic tissues

In some respects, the classical model system *D. melanogaster* is a poor representative of insects and *T. castaneum* is a good model system to study such processes. For instance, insects evolved extraembryonic membranes as an adaptation to life on land. In most insects (including *T. castaneum* but not *D. melanogaster*), the serosa forms a defensive layer around the embryo, protecting the growing embryo from dehydration (serosal cuticle) and invading microorganisms (serosal expression of immune genes) [44]. Formation of this extraembryonic layer necessitates additional morphogenetic movements that internalize the embryo and form the amnion, the other extraembryonic tissue found in insects. At the end of embryogenesis, this internalization (anatrepsis) becomes reversed (katatrepsis) and extraembryonic tissues are eliminated in the "dorsal organ". The mechanics of these movements are intriguing and in *T. castaneum* they are directly accessible to analysis by genetics and live imaging [45–47].

### Head development, brain evolution and behavior

The head of adult flies is highly derived, and even more so is the involuted head of fly larvae. Beetle larvae, in contrast, have an insect-typical head capsule with well-formed head appendages. How the insect head morphology comes about during development was first understood in *T. castaneum* [48, 49]. While many head (and brain) genes are widely conserved [48], there is much variation in head development between beetle and fly, some of which may be causally related to changes in head morphology [49, 50]. The brain of fly larvae is also strongly reduced, lacking a *central complex* until metamorphosis, while many insects including beetles show a reduced larval form of that structure [51]. Studies of embryonic patterning of the central complex in *T. castaneum* provide the basis for understanding evolutionary shifts of developmental timing in the brain [52–54]. Likewise, *T. castaneum* shows a more ancestral state with respect to the neuroendocrine system and the subesophageal ganglion has been studied with respect to adult neurogenesis [55–57]. Since the *T. castaneum* CNS is also amenable to systemic RNAi [58, 59], there should be a future for behavioral genetics via RNAi in the beetle, for example to study circadian activity or in death feigning behavior [60, 61].

### Appendage development and regeneration

Segmented appendages are the defining character of arthropods, and it is unfortunate that formation of these important structures is still incompletely understood. *D. melanogaster* research has identified the early acting appendage "gap genes" *Distal-less, dachshund* and *homothorax*, whose role is conserved in *T. castaneum* [62–64]. However, how the size and number of podomeres, and the position of joints are specified by the early acting appendage genes is not known. The fact that legs form during embryogenesis in *T. castaneum* allows studying the process with simple standard tools while studying leg formation during fly metamorphosis requires clonal analysis or transgenic RNAi. Given the genome-wide RNAi screening opportunities in *T. castaneum,* this model is predestined to study appendage segmentation in arthropods.

While legs arise in most insects as cylindrical outgrowths of the embryonic epidermis, in *D. melanogaster* limb formation is delayed and occurs within invaginated and flattened imaginal discs. Hence, the comparison of the genetic networks between fly and beetle should reveal how the derived state of fly limb development evolved [63]. Another process that is amenable to genetic analysis in *T. castaneum* is the ability of larval appendages to regenerate between larval molts [65].

### Metamorphosis and development of adult shape and organs

Many genes essential for postembryonic development of pupae and adults also function during embryogenesis. Systemic RNAi makes is easy to knock down an essential gene during metamorphosis without killing the embryo as would happen with a null mutation. Applying dsRNA to late larvae or pupae has revealed many late gene functions in hormonal regulation, the formation of adult limbs and wings, or other organs including hard adult cuticle, adult flight musculature, development and operation of defensive glands, or the development of gonads and gametes [2, 66–71].

### Population studies and physiology

Microevolutionary studies are actively pursued by a substantial part of the *Tribolium* research community (for review see [1]). In addition, several questions concerning physiological adaptations can be genetically analyzed in *T. castaneum*, including the use of defensive odoriferous glands producing toxins without poisoning the carrier [70]. Moreover, the species’ impressive adaptation to drought make it an ideal model system to study this phenomenon e.g. by studying the cryptonephridial organ [72]. Further, the life-span is considerably longer than that of *D. melanogaster* providing the opportunity to find additional anti-aging related processes.

### Pest management, systemic RNAi and selfish genetic elements

As a major pest of stored products, *T. castaneum* has long been used for investigating pest management and insecticide resistance [81]. While earlier studies focused on mechanisms and spread of pesticide resistance, recent interest has been building for using *Tribolium* as a model system for testing dsRNA-based pesticides in beetles and other insects. In RNAi mediated pest control, dsRNAs that target essential genes of pest species are sprayed onto plants, or produced by transgenic crops [82, 83]. Upon feeding on the crop, the dsRNA induces RNAi in the pest leading to its death. So far, *T. castaneum* has served as a screening tool for identifying the most efficient target genes [84, 85]. In the future, it might be useful for optimization of target sequences and to study RNAi dynamics. Since the capability to induce RNAi systemically varies widely among beetles and other pest species, the mechanism of dsRNA uptake and spread has also come into focus, which can be thoroughly studied in *T. castaneum* [86, 87].

A most intriguing example of selfish DNA was discovered by RW Beeman in *T. castaneum*, causing skewed segregation ratios in genetic crosses, named MEDEA (Maternal-Effect Dominant Embryonic Arrest, [88]). This element can spread in beetle populations through vertical transmission, by killing all offspring that do not inherit the MEDEA element. The principle underpinning MEDEA, consisting of a maternally expressed toxin and a zygotically expressed antidote, has inspired artificial selfish constructs that should similarly spread in natural populations and could be used for species-specific suppression of pest and vector species [89].

## Experimental approaches

### The workhorse technique: robust and systemic RNA interference

Gene knock-down via RNAi is an exceptionally robust and efficient technique in *T. castaneum* [90, 91]. The penetrance of phenotypes is usually close to 100% (i.e. all injected animals and all their offspring usually show a phenotype), and phenotypic strength comparable to the Null-phenotypes of genetic mutations are observed in most cases, e.g. for the Hox gene *Tc-sex combs reduced* [9], the patterning genes *Tc-Krüppel* and *Tc-knirps* [37, 92] and *Tc-Distal-less* [62, 91], as well as the eye color enzyme *Tc-vermillion* [93]. All the tissues and processes tested so far are amenable to RNAi, including physiological processes like cuticle [94] and pigment formation [95], immunity [96, 97] and neuroendocrine processing [56]. Development has been studied with respect to embryonic [17, 22] and postembryonic patterning of the epidermis [98], extraembryonic tissues [99], brain [53] and muscle formation [100].

Importantly, the RNAi effect is environmental, i.e. extracellular dsRNA is taken up by cells and triggers silencing. Hence, after injection into the body cavity, dsRNA is distributed via the hemolymph and appears to be taken up by all cells in the entire organism. This was convincingly shown when EGFP expressed from a ubiquitous promoter was silenced in all tissues [58]. We are not aware of tissues or stage that would not be amenable to RNAi. Hence, injection into the body cavity of any life stage leads to gene knock-down in most if not all cells. Embryos can be injected at late stages, leaving earlier gene functions unaffected, and injection into L5 or L6 larvae is often used to obtain phenotypes affecting metamorphosis. The RNAi effect is even transmitted from mother to offspring, such that injection into female pupae or adults leads to knock-down in the offspring as well [91]. The strength of silencing can be adjusted either by titrating the concentration of the dsRNA injected, or by collecting embryos at different time points after injecting their mothers [91]. dsRNAs are also taken up by cells in culture [101]. The molecular basis of dsRNA uptake remains enigmatic in *T. castaneum* but it seems to involve Clathrin-dependent endocytosis [86, 102]. Trials with oral uptake of dsRNA have met divergent success in different labs [103]. Whether RNAi silencing can spread from cell to cell (i.e. systemic RNAi sensu stricto) remains to be tested. In summary, virtually every gene in every tissue can be expected to be strongly silenced upon injection of dsRNA into any stage of *T. castaneum*.

### Cost- and time-efficient RNAi screening for unbiased detection of gene function

An outstanding strength of *T. castaneum* is the ease of large- or mid-scale RNAi screens. As part of the *iBeetle* screen [28, 29], a genome-wide collection of templates was produced, based on which dsRNAs can be commercially ordered, obviating the need for cloning (Eupheria Biotech, Dresden). This resource, together with the ease of microinjection (students learn the procedure within a day), enables mid or large scale RNAi screens to search for novel gene functions or to test gene lists derived from omics analyses. In our experience, the upper limit of the throughput is about 100 genes per week per screener (including injection, processing and screening for a simple phenotype like lethality). The *iBeetle* screen was a particularly complex screen because many different types of phenotypes were scored in parallel, resulting in relatively low throughput of 21 genes per week per screener. Based on these experiences, testing for example all transcription factors (around 800 genes) would take between 8 and 13 weeks depending on the complexity of the readout. Thus, *T. castaneum* is an excellent system for quickly testing the function of many genes, e.g. based on omics candidate gene lists.

### Elaborate and expanding transgenic toolkit

*T. castaneum* was the first species where the 3xP3 eye marker, based on an artificial Pax-responsive enhancer, was successfully applied [104]; 3xP3 has become a standard element of transgenic constructs in many insects. Besides the *piggyBac* transposon, other transposable element systems like *Minos* [105] or maize *Ac/Ds* (Distler & Klingler unpublished) work as transgenesis vectors as well. Transgenesis is performed as in *D. melanogaster,* by injecting early blastoderm stages with a plasmid containing transposon sequences flanking the cargo, mixed with a helper plasmid or mRNA that serve as a source of the transposase. The efficiency of transgenesis is similar to that using the P-element in *D. melanogaster*, and the genomic distribution of insertions is quite random [106]. Heat-shock and UAS/Gal4 mediated gene misexpression have been established [107, 108], the activity of several enhancers has been tested [109] and dedicated in vivo imaging lines have been established [110, 111]. Of note, enhancers and core promoters taken from *D. melanogaste*r show poor activity in *T. castaneum,* such that the use of endogenous elements is recommended. Core promoters that have proved effective in enhancer trapping and in genetic constructs include the *Tc-hsp68* and the super core promoter 1 (SCP1)-the latter being active in both *D. melanogaster* and *T. castaneum* [109]. Cre-Lox mediated recombination is working efficiently [111] opening the way for flip-out technologies like brainbow [112].

The identification and characterization of enhancers driving specific patterns is the limiting step in many applications. Several enhancers expected to drive ubiquitous expression such as *Tc-alpha-tubulin*, *Tc-EF1-alpha* and *Tc-poly-ubiquitin* appear not to be active in all cells at all stages and often show patchy or mosaic expression (Averof and Bucher labs, unpublished observation). A number of efforts testing putative developmental enhancers by placing them in front of reporter genes have failed. The likelihood of identifying active enhancers is improved by taking into account chromatin accessibility and using additional bioinformatics predictions [109]. The *cis*-regulatory elements of ubiquitously expressed genes are often located within few kilobases upstream of the transcription start site.

### Forward genetic screens-from irradiation to transposon mutagenesis

**Fig. 2:**
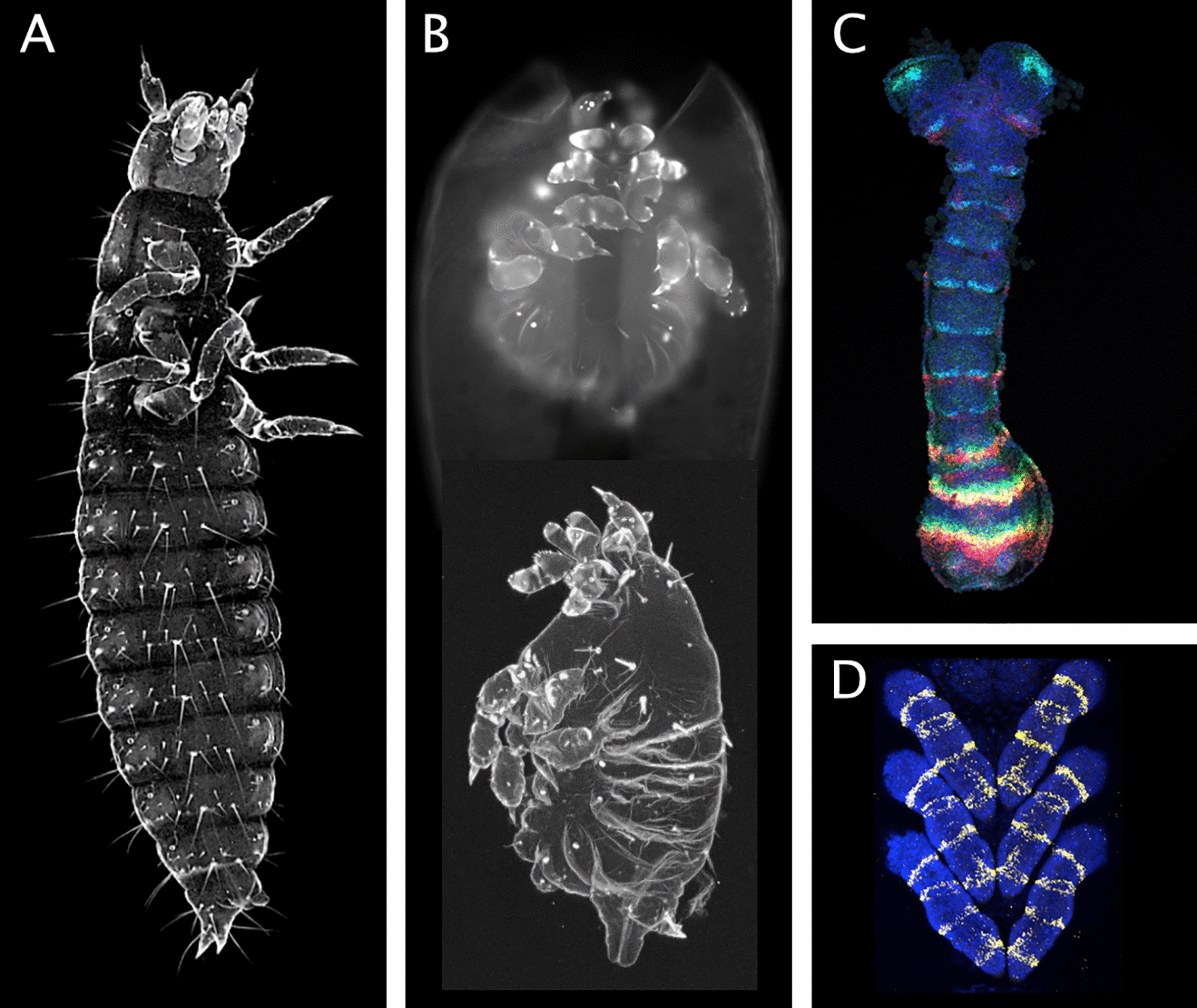
Cuticle phenotypes and in situ stainings. **A**, **B** Cuticle autofluorescence images of a wildtype 1^st^ instar larva (**A**) and two larvae homozygous for a transposon insertion into the first exon of the *Tc-cycD* gene. Reduced cell proliferation results in smaller embryos with shortened thoracic appendages (**B**). (**C**, **D**) Hybridization chain reaction staining of the pair rule genes *Tc-runt* and *Tc-odd-skipped* together with *Tc-wingless* as segmental marker in an elongating germ band (**C**) and of *Tc-odd-skipped* in the embryonic legs (**D**). Anterior is up in all panels. The larva in (**A**) is 1.4 mm in length, the embryo in (**C**) is 0.87 mm long. Image (**C**) by Felix Kaufholz, (**D**) by Christine Zellner

The relatively short generation time of *T. castaneum* allows performing classic forward mutagenesis screens. Irradiation and chemically induced mutagenesis were used to generate homeotic mutations that allowed to identify the single HOX complex [42]. A screen based on chemical mutagenesis led to the identification of pair-rule and gap-gene mutants [34, 37, 92, 113] and tools for parallel processing of up to 25 single pair matings have been developed [114]. The mobilization of piggyBac transposons has been applied for large-scale insertional mutagenesis and enhancer trapping in the *GEKU screen* [106] (Fig. 2). Based on mutants and molecular markers, a genetic map was generated, which was crucial for guiding a chromosome level assembly of the genomic sequence in times prior to long read sequencing [76, 78, 79].

Given the challenge of characterizing the often complex mutations induced by irradiation, the difficulty of mapping mutations and the effort of keeping such lines, we think that the use of transposon-mediated screening remains the most convenient forward genetics option, since the transposon marks the mutated locus and facilitates stock keeping based on a visible eye marker. However, given the effort it takes to reach saturation in genetic screens and the subsequent manpower needed for stock keeping, forward genetic screens seem less attractive. Hence, instead of performing forward genetic screens, approaches using large scale RNAi screening and genome editing to generate specific mutants may be more convenient for asking most questions.

### Genome editing for transgene insertion, gene modification and clonal cell marking

Genome editing based on CRISPR/Cas9 is highly efficient in *T. castaneum,* with knock-out efficiencies including mosaicism in > 50% of the injected animals and a high rate of germline transmission [115]. Knock-in of constructs using non-homologous end joining works reasonably well, with 0.1 to 10% of injected animals having at least one transgenic offspring. Constructs for generating targeted enhancer trap lines using this technique are available [53, 116]. Homology directed repair is efficient when replacing the EGFP of a transgene with another fluorescent protein [115], but less efficient when replacing larger stretches of DNA of essential genes (Bucher, unpublished results). Part of the problem may be the lethal effects of somatic mutations, which could be overcome with a transgenic line restricting expression of Cas9 to the germline. Efforts to generate such lines are underway (Gilles, Schinko, Bucher, personal communication) [117]. A system to enable stochastic clonal cell marking and clonal analysis of gene functions (Valcyrie) based on CRISPR/Cas9 driven excision has been established, opening interesting applications [118].

### In vivo* imaging and biophysics*

Markers for live imaging based on injected mRNA or transgenic lines (reporter constructs and enhancer trap lines generated by random insertion or CRISPR approaches) are available, providing a global view of embryonic development, including patterns of cell proliferation, morphogenetic movements, cell migration and innervation, enabling the analysis of the underlying biophysical mechanisms [45, 110, 116, 119–121].

### Genetic mapping

Surprisingly few QTL and GWAS studies have been conducted in *T. castaneum* so far [73–75], even though SNPs are frequent, and other mapping markers have been available for some time [76–78]. Frequently used strains are the San Bernardino (SB, mainly in the European evo-devo community-originally obtained from A. Sokoloff by K. Sander), Georgia (GA) and cSM strains (in the USA). An inbred line derived from GA was used for genome sequencing (GA2) [79, 80]. An India-derived strain (Tiw-1) has been used for genetic mapping experiments in combination with SB and other American strains, as its genetic distance from most other strain offers plenty of molecular markers (about one SNP per 200 bp when compared to SB) [37, 76, 77].

## Research community and resources

The *iBeetle*-Base (http://ibeetle-base.uni-goettingen.de/) provides gene sequence information, links to respective *Drosophila* homologs, and phenotypic information from the *iBeetle* RNAi-screen [28, 122, 123]. Further, it allows users to download the sequences of the entire genome, individual genes or coding sequences, and includes a genome browser. The *T. castaneum* morphological ontology *TrOn* describes a machine-readable semantic representation of morphological terms and their definitions [124]. An extensive transcriptomics database similar to the FlyAtlas [125] is being established (BeetleAtlas; https://motif.mvls.gla.ac.uk/BeetleAtlas/; K.V. Halberg, personal communication).

The first genome sequence included genetic mapping data [79] and was enhanced by re-sequencing and by adding extensive RNA-seq data [80]. An assembly based on long read technology is in the making.

dsRNAs targeting any set of *T. castaneum* genes can be ordered from Eupheria Biotech GmbH, Dresden.

A community mailing list is open for discussion of technical questions and is also used for announcing meetings and courses (subscribe at https://listserv.gwdg.de/mailman/listinfo/T. castaneum-community). Community meetings, which are often attached to the EuroEvoDevo (EED) meeting, are announced via this list.

## Data Availability

Contact the authors for wildtype or transgenic strains and constructs.

## Abbreviations

RNAi: RNA interference
dsRNA: Double stranded RNA
UAS/Gal4: Binary expression system
Cre/Lox: Recombination system
FFP1: Recommended level of protection for face masks
CNS: Central nervous system
CRISPR: Clustered Regularly Interspaced Short Palindromic Repeats—a system used for genome editing
Cas9: Enzyme required for genome editing
EGFP: Enhanced green fluorescent protein
Ac/Ds: Maize transposable system
GEKU screen: Labs from Göttingen, Erlangen, Kansas and USDA performed this insertional mutagenesis screen
*Tc-hsp68*: Heat-shock-promoter 68 in *Tribolium*
